# A Narrative Commentary on the Use of a Rational Emotive Behavior Therapy-Informed Group to Address Irrational Beliefs, Posttraumatic Stress Disorder, and Comorbidities

**DOI:** 10.3390/brainsci14020129

**Published:** 2024-01-26

**Authors:** Allen B. Grove, Brooke A. Green, Savannah M. Kaye, Christina M. Sheerin

**Affiliations:** 1 Central Virginia Veterans Affairs (VA) Health Care System, 1201 Broad Rock Blvd, Richmond, VA 23249, USA; 2 Virginia Institute for Psychiatric and Behavioral Genetics, Virginia Commonwealth University, Richmond, VA 23298, USA; christina.sheerin@vcuhealth.org

**Keywords:** PTSD, irrational beliefs, REBT, depression, anxiety, Veteran

## Abstract

Irrational beliefs of Demandingness, Catastrophizing, Low Frustration Tolerance, and Depreciation have demonstrated prevalence in disparate areas of life, including psychopathology, the military, politics, religion, and education. Individuals with mental health concerns, such as Post-Traumatic Stress Disorder (PTSD), endorse elevations in such thoughts compared to the general population. This commentary describes the rationale for focusing on irrational beliefs in efforts to address PTSD and presents the Rational Emotive Behavior Therapy (REBT)-Informed Group for PTSD as a potential novel application of a well-established intervention. In support of these suggestions, we present a narrative review of the published work on irrational beliefs and REBT tenets as relevant for PTSD. We then introduce and describe the REBT-Informed Group intervention, summarize the prior preliminary research conducted by our group, and present some novel data from a re-analysis of this prior work. We end with commentary related to future directions of REBT approaches for PTSD to address limitations and expand the impact of the treatment to military and other Veteran or civilian populations.

## 1. Introduction to Irrational Beliefs

Irrational beliefs are defined as conscious or preconscious thoughts that are illogical, extreme, or rigid [1,2]. There are four primary types: “(1) Demandingness (i.e., ‘absolute’ statements often involving ‘should, must, have to, need to’), (2) Catastrophizing (i.e., statements describing things as ‘awful, terrible, horrible, the worst’), (3) Low Frustration Tolerance (i.e., statements such as ‘I can’t stand it’), and (4) Depreciation (i.e., overgeneralization of negatives, disqualification of positives)” ([3], p. 218). Such beliefs often lead to emotions, such as anxiety, depression, anger, or guilt, and other psychopathology [1,4,5,6,7,8,9,10]. The following is a narrative review and commentary on the potential utility of Rational Emotive Behavior Therapy (REBT) for irrational beliefs in civilian and military contexts, focusing, in particular, on Post-Traumatic Stress Disorder (PTSD) [3,7,11].

## 2. Presence of Irrational Beliefs in Select Domains

Below, we highlight the domains in which irrational beliefs may present themselves, along with examples of such beliefs associated with each of the four types.

### 2.1. Irrational Beliefs in PTSD

Irrational beliefs are notable in people with PTSD [7,8,9,12], which is typified by re-experiencing symptoms (e.g., nightmares and intrusive memories), hyperarousal (e.g., concerns about having one’s back to others and alertness while being in a crowded restaurant), avoidance (e.g., staying away from people, places, things, or situations that increase other PTSD symptoms), and negative alterations in cognitions or mood (e.g., unhelpful thoughts about oneself, others, the world, or the future and perceiving that one is detached, distant, or numb) [13].

General irrational beliefs create vulnerability for the development of trauma-specific maladaptive cognitions following exposure to trauma [12]. Overly negative interpretations of traumatic experiences are influenced by pre-existing schemas that increase vulnerability to maladaptive beliefs about oneself, others, and the world [14]. Irrational beliefs formed during or after traumatic events can lead to inadvertent “*current* cognitive and behavioral re-traumatizing” because they are actively constructed and maintained (sometimes years later) by those who have gone through such situations ([2], p. 226), and they often lead to emotions such as anxiety, depression, anger, or guilt [1,4,5,6,7,8,9,10]. Ellis [15,16] posited that survivors of trauma can experience primary and secondary irrational beliefs, with primary irrational beliefs pertaining to the assessment of the traumatic event (e.g., “I should’ve fought harder against my attacker”) and secondary irrational beliefs encompassing those about the aftereffects of the trauma (e.g., “It means I’m weak because I have PTSD”; or “If I let myself feel my emotions, I’ll lose my mind”). A high level of irrational beliefs is associated with avoidance, re-experiencing symptoms, and other trauma-related sequalae [17]. Negative alterations in thoughts, especially safety, trust, and esteem-related cognitions, are hallmark symptoms of PTSD [18], with attentional biases toward stimuli perceived as threatening, potentially leading to hyperarousal symptoms [12]. These maladaptive and irrational beliefs can exacerbate other classic symptoms of PTSD, such as guilt or anger [10].

DiGiuseppe et al. [19] and Hyland et al. [9] posited that Demandingness beliefs are at the core of psychological disturbance. Hyland et al. [9] demonstrated the critical impact of this irrational belief for those with PTSD, with Demandingness being associated with each cluster of symptoms via secondary irrational beliefs. People with PTSD may experience cognitions such as “I should’ve known he was dangerous”, or “I should’ve been able to save my battle buddy” [20]. In addition, Catastrophizing beliefs, such as “I’m a horrible monster because of what I’ve done,” can result in shame, anger, and guilt [10]. The cognitive error of Depreciation is illustrated by beliefs such as “I’m broken now because I have PTSD,” or “Having PTSD means I’m weak” [18]. Self-stigma appears to pose a challenge for those with PTSD [21,22,23,24], with one study showing that self-stigma was more prevalent among Veterans with PTSD than those with other psychiatric diagnoses [25]. In addition, research has indicated that Low Frustration Tolerance beliefs (e.g., “If I let myself feel my emotions, I’ll lose my mind”) also contribute to and exacerbate symptoms of PTSD [26,27], as these individuals may be more inclined to engage in maladaptive avoidance behaviors than those who do not experience such thoughts [28].

### 2.2. Irrational Beliefs in the Military

Military service may create a unique vulnerability for the development of irrational cognitions in the setting of combat or other military-related trauma, given the military’s emphasis on rigid beliefs in the context of life-or-death experiences [3,7]. While inflexible beliefs, such as “Mistakes are intolerable”, may be helpful in combat or other high-risk scenarios, these same beliefs may result in psychological distress, interpersonal conflict, and occupational challenges in post-military civilian life [9,12]. Similarly, an emphasis on “the mission always comes first” and that “anything less than perfect is failure” may lead to esteem-related challenges and unrealistic expectations of the self and others in the long term [21,24,29]. Additionally, catastrophizing or planning for the worst may be protective in a dangerous and threatening environment [7]; however, doing so outside of the parameters of combat may make one vulnerable to unnecessary anxiety, tension, and worry [10].

Low Frustration Tolerance is an issue in the military as well: “I can’t stand it when my superior treats me that way!” is a thought that many service members describe having, potentially leading to depression or anger [10]; however, they are, in fact, “standing it,” even if they are not consciously aware of this [11]. Service members also struggle with Depreciation. For example, soldiers, sailors, airmen, or Marines may think “Things never go my way!” or “I don’t get a cookie just because I did what I’m supposed to do!” Such thoughts often lead to depression [3,4,7,10]. Given the stigmatization of PTSD evident in the military population [30], maladaptive esteem-related beliefs can be compounded by ostracization from one’s unit or peers if those individuals also overgeneralize negative attributions (i.e., unreliable, untrustworthy, etc.) upon learning of a fellow service member’s struggles with PTSD [21,24].

### 2.3. Irrational Beliefs in Select Civilian Contexts

#### 2.3.1. Irrational Beliefs in Politics

Irrational beliefs may be on the rise in the United States and around the world, as evidenced by a notable degree of political polarization and extreme socio-political beliefs [31]. This extreme polarization can create vulnerability to totalitarianism and an intolerance of differing opinions, which are conditions linked to irrational beliefs [31]. Radicalization, or the development of extreme behaviors, thoughts, and emotions, typically involves prioritizing the rights of one group of individuals over the rights of others, often in response to the perception of unfairness or injustice. In response to such a belief, people may angrily demand that they be met with a fair world (an irrational belief in itself) [32] and expect “promises” that injustices will not reoccur [33]. These thoughts may include Demandingness beliefs, such as “I should be able to spend my hard-earned money on my family instead of paying taxes for services I don’t use,” or Demandingness/Depreciation beliefs, such as “Wealthy people are undeserving of the success they have and should reallocate more of their resources.”

Although irrational beliefs are stereotypically prescribed to conservative political causes or leaders, absolute beliefs may be equally problematic within liberalism [31]. Demandingness beliefs are evidenced for both sides by an overemphasis on “Utopianism,” or on the way the world “should be” as opposed to what it is in reality [31]. Political discourse may include Demandingness thoughts, such as “People who disagree with me must be stopped!” or “All people who vote for the other party must be crazy!” ([7], p. 8). People are often able to notice such irrational beliefs when “the other side” describes them, but they are much less able to notice them when the “good” side describes them [34]. Such thoughts often lead to anxiety or anger. Catastrophizing is common within political discourse. For example, “It would be awful if the other side wins this election!” or “The world has gone to hell in a handbasket!” are the kinds of thoughts that might lead to anxiety or anger and potentially depression if “the other side” actually won the election. Low Frustration Tolerance has also become common in the political world: “I can’t stand it when [particular politicians from the other side] speak!” or “[Insert leader’s name here] is terrible/no good/awful for this country.” Such cognitions may lead to anger. Depreciation is regularly associated with political discussions as well: “Nothing remotely good ever comes from the other side!” Such thoughts may lead to anger or depression [4,7,10,31].

#### 2.3.2. Irrational Beliefs in Religion

Religion may also be a source of irrational beliefs. Several authors (e.g., Johnson [35], Nielson and Ellis [36]) have noted that REBT was often criticized by religious leaders as it appeared to espouse “moral relativism” when there is only one truth, according to such leaders. For example, one study examining irrational and extremist beliefs in Jordanian prisoners sentenced for terrorism or political, religious, or social extremism found that some of the most commonly held beliefs by the participants included “musts” (i.e., Demandingness thoughts) [37]. Most religions demand that people “should” or “should not” do certain things (e.g., lie; steal; eat red meat or eat it at certain times of the day or month; wear particular clothing; commit adultery; kill; etc.). It is not difficult to imagine that if the above prohibited behaviors occur, Catastrophizing (e.g., “It is awful that I did that!”), Low Frustration Tolerance (e.g., “I can’t stand it when others do that!”), or Depreciation (e.g., “There is nothing good about me because I did that!”) are then likely to follow, further impacting anxiety, depression, anger, or guilt [4,7,10].

#### 2.3.3. Irrational Beliefs in Academia

Educational institutions and the field of psychology itself are often sources of irrational beliefs (e.g., [38,39]). People who express views outside those of the majority in educational institutions (including psychology departments) are sometimes ostracized or banned from speaking because they are not saying what the majority suggests they “should” say or what students or faculty at such places of learning “should” hear [40,41], which is consistent with the Demandingness of politics and religion above. It is again not difficult to imagine that if the above contrarian expressions or behaviors occur, Catastrophizing (e.g., “What they are saying is awful!”), Low Frustration Tolerance (e.g., “I cannot stand it when they say that!”), or Depreciation (e.g., “There is nothing good about that person!”) are likely to follow, again leading to anxiety or anger [4,7,10].

## 3. Rational Emotive Behavior Therapy (REBT)

Rational Emotive Behavior Therapy (REBT) is a cognitive- and behavior-based treatment that was first developed by Albert Ellis in the 1950s to increase the speed of improvement compared to the years patients spent undergoing traditional psychoanalysis or psychodynamic treatment [1]. REBT focuses on the incorporation of thinking, feeling, and behaving to provide a holistic approach to the treatment of psychological disorders [42] and has been called the original Cognitive Behavior Therapy (CBT) [43]. Specifically, the goal of REBT is to assist clients in identifying and addressing irrational philosophies (i.e., Demandingness, Catastrophizing, Low Frustration Tolerance, and Depreciation) that lead to distress and dysfunction and replacing these beliefs with ones that facilitate well-being [2]. The theory of REBT suggests that symptoms of anxiety and depression stem from irrational beliefs individuals hold about themselves, others, and the world (please see the above sections for examples), as well as their thoughts, behaviors, or emotions when these beliefs are unfulfilled [4,10].

As described above, irrational beliefs are prevalent among individuals with PTSD, and these beliefs, in conjunction with traumatic incidents, perpetuate symptoms and can result in maladaptive coping [15]. REBT’s approach for treatment is unique from other cognitive therapies as it focuses primarily on these irrational beliefs to mitigate the consequences that engaging in these beliefs create for individuals with PTSD (e.g., anxiety, depression, anger, and guilt) [10,11]. In addition, as described by Ellis [2], Grove et al. [7], and Matweychuk et al. [43], REBT is different from Cognitive Therapy (CT) in other areas: it stresses the worth of the individual as a person (regardless of whether one thinks, behaves, or feels in a “rational” way) and helps clients rate “situational” behaviors (as opposed to relatively “global” behaviors rated in CT, e.g., “That was a dumb thing I did,” rather than “I am dumb”).

### Relevance of REBT for PTSD

For those with PTSD, irrational beliefs may result in avoidance of community and increased isolation, and may inadvertently lead to an increase in irrational beliefs themselves [3,4,7,10]. Avoidance, which may include withdrawing from others, is a prominent symptom of PTSD [13]. In fact, of the difficulties associated with PTSD, avoidance is more likely to contribute to the maintenance of PTSD [44,45] than any of the other core symptoms of the disorder (i.e., re-experiencing, hyperarousal, and negative cognitions/emotional numbing) [13]. Irrational beliefs, such as “I’m not safe anywhere” or “No one can be trusted or be safe to be around,” may lend themselves to behavioral avoidance. If those with PTSD are struggling with avoidance and an increase in irrational beliefs exacerbates such difficulties, people with PTSD may benefit from a treatment that can reduce irrational beliefs.

Trauma-focused treatments, such as Cognitive Processing Therapy (CPT) [18], Prolonged Exposure (PE) therapy [46], and Eye Movement Desensitization and Reprocessing (EMDR) therapy [47], are the most highly recommended treatments for PTSD. Of these treatments, only CPT focuses on irrational beliefs directly, despite the variance explained in PTSD by such cognitions [7,9]. While PE, CPT, and EMDR have been shown to be efficacious treatments for PTSD [48,49,50,51], the effect sizes for these frontline, evidence-based, trauma-focused treatments for PTSD remain modest [52]. Thus, there remains a need for effective non-trauma-focused interventions, particularly given the importance of patients’ choices [51], including the preference for non-trauma-focused treatments for those who have concerns about their ability to tolerate this relatively intensive form of treatment [53] and efforts to reduce treatment dropout [54].

## 4. The Rational Emotive Behavior Therapy (REBT)-Informed Group

The development of the REBT-Informed Group intervention grew out of (1) an identified clinical need [3] including non-trauma-focused treatments for PTSD [53], (2) the established association of irrational beliefs to psychiatric distress [1,4,6,7,8,9,10], and (3) the recognition of the relevance of irrational beliefs for PTSD populations in particular [7,11,12]. The REBT-Informed Group may be an intervention that is particularly suited to psychiatric conditions in which irrational beliefs are prominent, such as PTSD [3,7,11]. The group was developed by the first author over 13 years of clinical practice and has been used with active-duty military and Veteran populations in clinical settings. The REBT-Informed Group was initially adapted from the work of Burns [55] with a significant focus on CT methods for treating anxiety and depression. The group continues to include many aspects of CT (e.g., control, mind reading, and fortune telling, hence the name “REBT-Informed Group” rather than “REBT Group”) but has evolved to substantially emphasize techniques of REBT theory [3,11,12,56].

As Grove et al. [3] noted, the REBT-Informed Group addresses several gaps in PTSD treatment. By including those with PTSD in a group format but focusing on anxiety and depression rather than PTSD itself, both self-stigma and treatment-seeking stigma may be reduced, given evidence suggesting that self-stigma may be more related to PTSD than to depression symptoms [21] and that those with PTSD report reductions in self-stigma when engaging with similar peers (e.g., other people with PTSD) [24]. The group emphasizes present concerns without processing traumatic memories, which may be a relatively “safe” initial option for people with high levels of avoidance [53] or as an initial foray into treatment. The content targets irrational beliefs, which may help increase cognitive flexibility in support of future trauma-related treatment (e.g., CPT) [18]. The brief treatment approach may be attractive to those with limited leave time from work or school and those with treatment-related stigma [57]. Moreover, the reduced number of sessions and the group format increase access to treatment [11,58].

### 4.1. REBT-Informed Group Structure

The group includes five sessions, as described in *Rational Emotive Behavior Therapy-Informed Treatment for Anxiety and Depression: Facilitator’s Guide* [11]. Session 1 provides an overview of the group and the group structure, increases patients’ understanding of anxiety and depression symptoms, normalizes the purposes of anxiety and depression, and offers hope that patients can learn skills to better manage anxiety and depression symptoms that interfere with their daily lives [32,59]. Session 2 focuses on increasing patients’ understanding of (1) potential causes of anxiety and depression symptoms; (2) how thoughts, behaviors, and emotions are related to one another; and (3) the concept of control and its relationship with anxiety and depression [2,11].

Session 3 provides an overview of common unhealthy thinking patterns, such as mind reading [55], fortune telling [44], Catastrophizing, and all-or-nothing thinking, and increases patients’ understanding of how these thought patterns affect anxiety and depression. Catastrophizing, Low Frustration Tolerance, and the first Demandingness thoughts (e.g., all, nothing, perfect, failure, right, wrong, etc.) are introduced, and their relationships with anxiety, depression, anger, and guilt are discussed. Session 4 reviews common unhealthy thinking patterns, including Demandingness, spending particular time on “should” statements, and disqualifying the positives. Therapists also have patients practice identifying unhealthy thinking patterns and changing their thoughts to be more balanced than they were previously. The patients are introduced to several more Demandingness terms (e.g., should, must, ought, need to, have to, etc.). The patients and the group leader discuss the concept of “shoulding on oneself” [2] and the resulting anger or guilt. Depreciation is also introduced in this session as patients and the group leader note that depressed people are “experts” at noticing negative things in their lives and are much less observant of positive situations [11].

Session 5 includes a review of the group material, an introduction to problem-solving steps, relapse prevention, treatment planning, and processing group termination. The patients and the group leader discuss stereotypes, society’s concerns that one “shouldn’t” stereotype, how such thoughts can be useful, and the importance of challenging them as well. This leads to a continued discussion of Depreciation and Demandingness thoughts and ways to reduce them. The patients and the group leader also discuss problem solving, with an emphasis on slowing down the process rather than making an “emotional” decision [55]. Finally, the patients and the group leader discuss other treatment options within the PTSD program (e.g., CPT) [18] as well as the patients’ thoughts and emotions about the completion of the group [11].

### 4.2. Prior Research on the REBT-Informed Group and Re-Analysis of Existing Data

The primary goal of the REBT-Informed Group is to help patients become aware of, challenge, and change their irrational beliefs, leading to a decrease in PTSD and other symptoms [3,12,60]. Program evaluation efforts on the REBT-informed intervention, as described above, have been conducted by our team to examine the presence of irrational beliefs in our clinical sample of combat Veterans, the effectiveness of an REBT-informed intervention to address PTSD and other mental health symptoms, and the impact of changes in irrational beliefs as a driver of symptom improvement. Here, we include a re-analysis of the existing data to provide new descriptive statistics of irrational beliefs, examine their correlations with PTSD cluster symptoms and *t*-test results regarding changes in specific irrational beliefs, as well as summarize relevant prior published findings.

**Figure 1:**
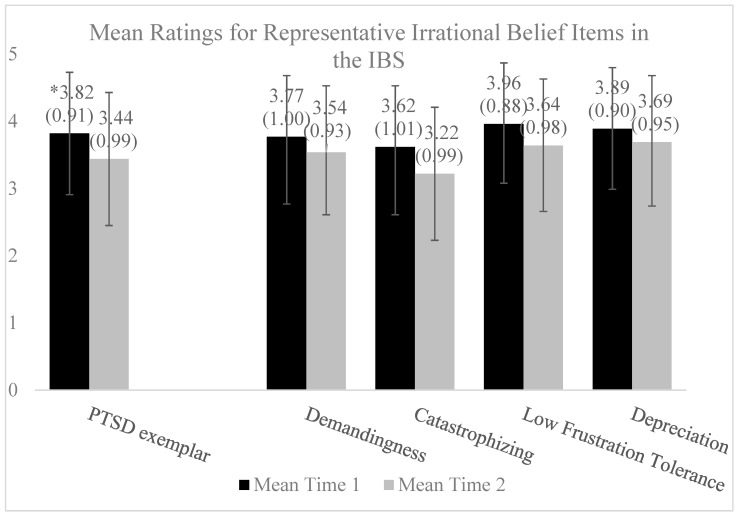
Mean ratings for representative irrational belief items in the IBS at baseline and post-intervention (*N* = 86). * The item response range is 1–5; means and standard deviations for each item and time point are presented in the figure. Exemplar items from the Irrational Beliefs Scale (IBS) include a frequently reported statement from those with PTSD: “Many events from my past so strongly influence me that it is impossible to change” (item 11); Demandingness: “Individuals who take unfair advantage of me should be punished” (item 7); Catastrophizing: “It is terrible when things do not go the way I would like” (item 8); Low Frustration Tolerance: “I cannot help how I feel when everything is going wrong” (item 10); and Depreciation: “To be a worthwhile person I must be thoroughly competent in everything I do” (item 1). Pre–post change was examined using paired-sample *t*-tests across individual items using Glass’ *delta* [64] as follows: PTSD-specific (*delta* = 0.42), a medium effect; Demandingness (*delta* = 0.23), a small effect; Catastrophizing (*delta* = 0.40), a medium effect; Low Frustration Tolerance (*delta* = 0.36), a small-to-medium effect; and Depreciation (*delta* = 0.22), a small effect [65].

Within our treatment population sample (please see [7] for detailed methods and sample information; demographic information is also available in Supplemental Table S1), we examined the total and individual item scores on a measure of irrational beliefs, the Irrational Beliefs Scale (IBS) [61]. The mean scores on the IBS were high (*M* = 72.52, *SD* = 10.25; possible range on the measure is 20–100) prior to beginning the intervention, demonstrating high rates of irrational beliefs in a PTSD clinical population [9,10,19]. These scores were, expectedly, higher compared to the established work with this measure in healthy populations (*M* = 61.78, *SD* = 11.13) [62] and similar, although slightly higher, compared to those of depressed samples (dysphoric group: *M* = 65.4, *SD* = 14.5; depressed group: *M* = 70.2, *SD* = 10.9) [63]. The mean scores were also newly examined for specific items in the IBS, selected as examples of the four types of irrational beliefs (i.e., Demandingness, Catastrophizing, Low Frustration Tolerance, and Depreciation), along with an additional statement particularly relevant for PTSD [44]. The mean scores for these exemplar items appeared to be similarly and highly endorsed in our sample. Finally, we also examined change through paired-sample *t*-tests in the exemplar items using Glass’ *delta* as a measure of the effect size out of concern that the treatment itself affected a combined standard deviation [64]. Each exemplar decreased, the effect of which varied from small to medium (see Figure 1).

In support of the theoretical prediction of REBT that irrational beliefs are indeed crucial in the development or maintenance of psychopathological symptoms [1,4,6,10], and to investigate whether they are relevant for PTSD in particular [7,8,9,12], we examined zero-order correlations in our sample between the IBS total score and PTSD cluster scores from the Post-Traumatic Stress Disorder (PTSD) Checklist for DSM-5 (PCL-5) [66,67] prior to group initiation. These correlations are presented in Table 1. As would be expected, the IBS was significantly moderately correlated with all four clusters, with the strongest correlation (*r* = .55, *p* < .001) being found with the cognitions and mood symptom cluster. Irrational beliefs were associated with 20.4% of the variance in re-experiencing symptoms; 19.4% in avoidance symptoms; 29.9% in negative cognitions/emotional numbing symptoms; and 24.9% in hyperarousal symptoms, demonstrating medium-to-large or large effects across the clusters [65]. These patterns are consistent with Grove et al.’s [7] suggestion that a large cognitive shift is not necessary to demonstrate subsequent notable declines in psychiatric symptoms and with the philosophy of this treatment that emphasizes diminishing but not eliminating irrational beliefs. These data align with the prior work by Hyland et al. [9] using structural equation modeling, wherein irrational beliefs were found to explain 67% of the variance in re-experiencing symptoms, 50% in avoidance symptoms, 67% in negative cognitions/emotional numbing symptoms, and 56% in hyperarousal symptoms.

Prior work by our group [3] demonstrated the effectiveness of the REBT-Informed intervention in a real-world clinical sample of military Veterans with PTSD (and other comorbidities, including anxiety and depression). In the treatment completers, significant reductions were found for PTSD symptoms as well as depression symptoms. Importantly, reductions in PTSD symptoms in the five-session REBT-Informed Group were comparable to a matched sample receiving a ten-session treatment-as-usual group for PTSD within the same clinic [44,53]. These findings were promising regarding the specific relevance of REBT for a Veteran PTSD sample and the benefit of a brief intervention. Following a demonstration of the effectiveness of the intervention, we sought to examine a core premise of REBT: irrational beliefs could be modified and a decrease in these beliefs would lead to a decrease in symptoms of PTSD, depression, and anxiety. This work [7] demonstrated that irrational beliefs significantly decreased from pre- to post-group for treatment completers and that a reduction in irrational beliefs predicted a significant decrease in PTSD, depression, and anxiety symptoms after controlling for several covariates (i.e., age; gender; race; ethnicity; employment status; relationship status; type of trauma; psychiatric medication use; and number of deployments). These findings lend support to the REBT theory, suggesting that a decline in irrational beliefs acts as a mechanism of change.

## 5. Discussion

As summarized in this paper, irrational beliefs of Demandingness, Catastrophizing, Low Frustration Tolerance, and Depreciation are pervasive both within the military [21,24,30] as well as civilian society in numerous domains [33,37,39,40]. These irrational beliefs are associated with both the development and maintenance of PTSD symptoms, along with comorbid anxiety and depression, as well as anger and guilt [1,2,3,4,6,7,8,9,10,12,44,56,60,68,69,70]. This suggests that a treatment addressing irrational beliefs may reduce PTSD symptoms as well as those of anxiety, depression, anger, or guilt for both military and civilian populations [8,10,12]. It is noted, however, that a limitation of this work by nature of a narrative review is the lack of a full, systematic review and a statistical analysis of what is available in the literature. Future work conducting a systematic review and meta-analysis of REBT-based interventions for PTSD as studies increase would be a useful next step to better understand its relevance and potential as a useful intervention for PTSD.

We propose REBT for PTSD treatment as a novel application of a well-established intervention [1,3,4,6,7,8,12,68]. Irrational beliefs are both transdiagnostic to numerous mental health conditions [10] and particularly relevant to PTSD [9], given that the symptoms are directly related to distorted beliefs/thoughts, as is recognized and addressed in other trauma-informed PTSD interventions (e.g., CPT) [18]. A unique aspect of addressing irrational beliefs in this manner may lie in the development or improvement of skills in recognizing, challenging, and changing these beliefs broadly, as is likely relevant and useful for focusing on the common comorbidities of anxiety, depression, anger, and guilt seen with PTSD [10,11]. Furthermore, these irrational beliefs are also addressed outside of the context of trauma-focused interventions [53]. Although this intervention may be sufficient for some, it may be a particularly useful adjunctive intervention prior to engaging in further trauma-focused work for individuals who continue to meet the criteria for PTSD (e.g., CPT) [18].

Despite the relevance of irrational beliefs to PTSD and the potential impact of addressing irrational beliefs [7,70], few treatments directly incorporating the challenge of irrational beliefs (e.g., CPT) [18] have been developed for trauma-focused (see [46,47]) or non-trauma-focused approaches [44,53]. We presented preliminary work demonstrating that REBT, including the REBT-Informed Group, is a promising intervention for PTSD [3,7,11] without directly addressing a previous traumatic event. However, the limitations of this work thus far include a small number of studies and a lack of randomized controlled trial studies to date. Thus, more stringent work is needed with larger and more diverse samples (e.g., other military populations (e.g., pre-9/11 combat Veterans, military sexual trauma, and active-duty military) and civilian populations) with PTSD symptoms and comorbid anxiety, depression, anger, or guilt to determine the effectiveness and generalizability in diverse groups. The expansion of the REBT-Informed Group could impact a considerable number of people suffering from PTSD and other mental health difficulties.

## Figures and Tables

**Table 1:** Correlations of IBS total score with PCL-5 subscale scores at baseline, prior to the REBT-Informed Group (*N* = 86).

	1.	2.	3.	4.	5.
1. Irrational beliefs	--				
2. Re-experiencing symptoms	.45 *	--			
3. Avoidance symptoms	.44 *	.49 *	--		
4. Mood and Cognitions	.55 *	.57 *	.56 *	--	
5. Hyperarousal symptoms	.50 *	.55 *	.53 *	.66 *	--

Note: * = *p* < .001; IBS = Irrational Beliefs Scale; PCL-5 = PTSD Checklist for DSM-5; REBT = Rational Emotive Behavior Therapy.

## Data Availability

All anonymized data, analysis codes, and research materials can be made available upon reasonable request from the corresponding author.

## Supplementary Materials

The following supporting information can be downloaded at https://www.mdpi.com/article/10.3390/brainsci14020129/s1, Table S1: Demographic and clinical information in REBT-Informed Group sample (*N* = 86).
