# Supplementary material for: A Narrative Commentary on the Use of a Rational Emotive Behavior Therapy-Informed Group to Address Irrational Beliefs, Posttraumatic Stress Disorder, and Comorbidities
Source: Brain Sci. 2024 Jan 26;14(2):129. doi: 10.3390/brainsci14020129 (PMC10886947; doi:10.3390/brainsci14020129)
Supplement: Supplementary file 1 [file brainsci-14-00129-s001.zip › brainsci-2759162-supplementary.pdf]

Supplemental Table S1. Demographic and clinical information in REBT-Informed Group sample ($N = 86$)

	***N* (*M*)**	**% (*SD*)**
Gender		
Female	8	9.30
Male	78	90.70
Race		
Asian American	1	1.16
Black/African American	54	62.79
Native American	1	1.16
White/Caucasian	25	29.07
Unreported	5	5.81
Ethnicity		
Hispanic/Latinx	9	10.47
Non-Hispanic/Latinx	76	88.37
Unknown	1	1.16
Age	44.60	7.65
Marital Status		
Married	45	52.33
Never Married	9	10.47
Divorced	12	13.95
Separated	6	6.98
Remarried	14	16.28
Employment Status		
Full-Time	47	54.65
Part-Time	1	1.16
Unemployed	12	13.95
Retired	17	19.77
Disabled	8	9.30
Non-Stable	1	1.16
Trauma History		
Combat	76	88.37
Combination of Traumas[a]	10	11.63
Taking Medications for PTSD		
No	20	23.26
Yes, currently	62	72.09
Yes, in the past	4	4.65

Number of Deployments	2.52	1.32

Note. [a]Combination of traumas includes combat as well as either childhood physical, sexual, or emotional abuse, adult physical abuse, or military sexual trauma (MST). PTSD = Posttraumatic stress disorder.
